# Role of hydrogen sulfide in endothelial dysfunction: Pathophysiology and therapeutic approaches

**DOI:** 10.1016/j.jare.2020.05.015

**Published:** 2020-05-19

**Authors:** Valentina Citi, Alma Martelli, Era Gorica, Simone Brogi, Lara Testai, Vincenzo Calderone

**Affiliations:** Department of Pharmacy, University of Pisa, via Bonanno n.6, 56125 Pisa, Italy

**Keywords:** Hydrogen sulfide, Endothelial dysfunction, H_2_S-donor, Cardiovascular diseases

## Abstract

**Background:**

The vascular endothelium represents a fundamental mechanical and biological barrier for the maintenance of vascular homeostasis along the entire vascular tree. Changes in its integrity are associated to several cardiovascular diseases, including hypertension, atherosclerosis, hyperhomocysteinemia, diabetes, all linked to the peculiar condition named endothelial dysfunction, which is referred to the loss of endothelial physiological functions, comprehending the regulation of vascular relaxation and/or cell redox balance, the inhibition of leukocyte infiltration and the production of NO. Among the endothelium-released vasoactive factors, in the last years hydrogen sulfide has been viewed as one of the main characters involved in the regulation of endothelium functionality, and many studies demonstrated that H2S behaves as a vasoprotective gasotransmitter in those cardiovascular diseases where endothelial dysfunction seems to be the central issue.

**Aim:**

The role of hydrogen sulfide in endothelial dysfunction-related cardiovascular diseases is discussed in this review.

**Key Scientific Concepts:**

Possible therapeutic approaches using molecules able to release H_2_S.

## Graphical abstract

**Figure:**
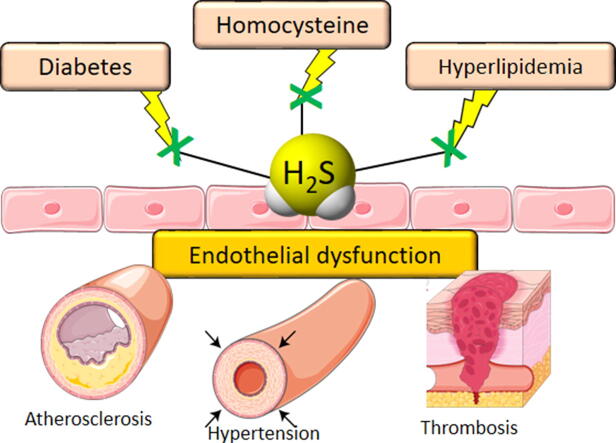

## Introduction

The vascular endothelium is a monolayer of dynamic cells which cover the inner surface of the entire vascular tree and it is known to be an important regulator of the vascular wall homeostasis. In particular, endothelial cells (ECs) represent both mechanical and biological barrier between the vessels and the underlying tissues through which continuous exchange of molecules between the interstitial tissue and blood occurs in the capillaries [1]. The endothelium is continuously exposed to shear stress due to the blood flow and such a mechanical stimulus triggers intracellular and biochemical signals which may alter the basal permeability [2]. Changes in the integrity of the endothelial barrier are related to different pathophysiological processes, including tissue remodeling, repair and inflammation [3]. In addition to its role as a barrier, healthy endothelium is involved in the maintenance of vascular tone, since it mediates smooth muscle relaxation through endothelium-derived hyperpolarizing factors, promotes antioxidant and anti-inflammatory effects through the inhibition of both leukocyte adhesion and migration and prevents smooth muscle cell proliferation and migration. The release and control of several endothelial bioactive molecules have been shown to counteract thrombosis and atherosclerosis [4].

The endothelial dysfunction (ED) refers to the loss of these physiological functions caused by different risk factors, including hyperglycemia, hypercholesterolemia and hyperhomocysteinemia (HHcy) [5]. ECs respond to such pathological conditions by increasing the levels of reactive oxygen species (ROS), and consequently enhancing the oxidative stress along the vascular tree. These conditions trigger several damaging events resulting in the inability of the endothelium to exert its fundamental activities, i.e the regulation of vascular relaxation and/or cell redox balance, leading to inflammatory response that usually promotes endothelial activation (inflammatory response), reduces nitric oxide (NO) production correlated with the impairment of vascular smooth muscle dilation [6].

Many evidence reported the vasoprotective effect of hydrogen sulfide (H_2_S) that is an endogenous gaseous molecule, synthesized in mammalian tissues from L-cysteine by two cytosolic pyridoxal-5′-phosphate-dependent enzymes: cystathionine β-synthase (CBS) and cystathionine γ-lyase (CSE) [7]. In addition, 3-mercapto sulfotransferase (3-MST) and cysteine aminotransferase (CAT) contribute to the biosynthesis of H_2_S [8]. Even though it has been long considered a toxic gas, recent studies described the fundamental role of H_2_S in the regulation of cardiovascular homeostasis; indeed, its deficiency is etiologically associated with several cardiovascular diseases [9].

In addition, H_2_S is also emerging as an essential molecule in controlling the homeostasis of endothelial function and an impairment of its endogenous production is related to the pathogenesis of ED [10]. Many studies demonstrated that H_2_S behaves as a vasculoprotective gasotransmitter by modulating different cellular pathways and interfering with a variety of vascular diseases. Indeed, H_2_S inhibits atherogenic modification of low-density lipoproteins (LDL) [11], prevents monocytes adhesion due to ECs activation [12], clearly promotes vasorelaxing responses [13], decreases intimal hyperplasia by inhibiting vascular smooth muscle cells (VSMC) migration and proliferation [14], limits vascular calcification [15], thrombogenesis and platelet aggregation [16], inhibits macrophage foam cell formation and degranulation [17], limits inflammatory responses and reduces plasma homocysteine (Hcy) levels in experimental animals [18].

Therefore, ED is widely associated with several pathological conditions including diabetes, hypertension, atherosclerosis and HHcy. The role of hydrogen sulfide in such ED-related diseases, is discussed, focusing the attention on the possible therapeutic approaches using molecules able to release H_2_S.

## Hydrogen sulfide and hypertension-related endothelial dysfunction

The correlation between ED and hypertension is mainly referred to as an alteration of the metabolism of nitric oxide (NO), due to a severe imbalance between the biosynthesis of vasodilator agents and the production of vasoconstrictor substances, which guarantee the control of vascular tone [19].

Indeed, among numerous vasoactive molecules, NO plays a crucial role in mediating vasodilation responses. Many studies demonstrated that the reduction of NO levels in vessels is associated with hypertension. The NO-deficiency impairs vascular homeostasis, alters the endothelium permeability and consequently leads to inflammatory and fibrotic processes, which are responsible for structural alterations, including vascular hypertrophy and increased vascular resistances. Consistently, chronic administration of NOS inhibitors causes sustained hypertension in several pre-clinical models [20], [21].

Besides NO, also the endogenous gasotransmitter H_2_S is emerging as one of the main characters in modulating the endothelium integrity and functionality. Numerous *in vitro*, *ex vivo* and *in vivo* studies demonstrated the vasoactive role of H_2_S, confirming the hypothesis that H_2_S importantly contributes to the regulation of vascular tone [22], [23]. In fact, like NO, H_2_S assures the maintenance of vascular homeostasis by the regulation of the complex balance of several components, including vasodilation responses as widely demonstrated for molecules able to release H_2_S [24], [25], [26], [27], [28], [29], [30], [31], [32], [33], [34], [35], [36].

The pathogenesis of ED-related hypertension seems to be also related to the reduction of H_2_S biosynthesis. Indeed, the downregulation of CSE, the enzyme which is responsible for H_2_S production in the vasculature, leads to the increase of blood pressure associated with the reduction of H_2_S bioavailability [37], [38], [39], [40]; accordingly, CSE-knock-out mice showed an abolished endothelium-dependent relaxation in resistance mesenteric arteries [23].

Furthermore, a human cohort study reported that hypertensive patients showed reduced H_2_S plasma levels [41], suggesting that H_2_S can be also viewed as a predictive biomarker for the development of high blood pressure [42]. The beneficial effects of H_2_S exogenous supply were highlighted by Xiao and colleagues, who reported that 20-week administration of NaHS – which is a molecule able to release H_2_S - lowered the arterial pressure and increased the production of NO, enhancing eNOS phosphorylation through the activation of peroxisome proliferator-activated receptor δ/ protein kinase B/ AMP-activated protein kinase (PPAR-δ/Akt/AMPK) signaling pathway [43].

H_2_S and NO share several beneficial effects in the cardiovascular system and many studies reported the mutual interaction between H_2_S and NO. Indeed, NO increases the uptake of L-cysteine, which is the main substrate of the H_2_S generating enzymes CSE and CBS, consequently leading to the increase of H_2_S production [44]; moreover, exogenous NO-donors trigger CSE activation in VSMC, increasing the biosynthesis of H_2_S [45].

Accordingly, the inhibition of eNOS attenuated the H_2_S-induced vasorelaxation in rat aorta [46] and inhibited the expression /activity of CSE in the cardiovascular system [47].

Coletta and colleagues observed that NaHS-mediated vasodilation was attenuated in eNOS-KO mice vessels [27] and, in accordance, NaHS increased the NO production through eNOS sulphydration in rat corpus cavernosum [48], [49].

The inhibition of NO synthase, using N(ω)-nitro-L-arginine methyl ester (L-NAME), induces ED-related hypertension, and is a widely used model for studying the effect of several vasoactive molecules, including H_2_S.

Indeed, in L-NAME-induced hypertensive rats, the lack of NO biosynthesis was associated with dysfunction of the cysteine/CSE/H_2_S pathway and exogenous administration of H_2_S prevented the development of hypertension [50].

Furthermore, H_2_S potentiates the NO-mediated vasorelaxing effect through the inhibition of phosphodiesterase-5 (PDE-5), prolonging the half-life of cGMP which is the key messenger in NO vasoactive signals [26].

However, another interaction has been hypothesized: NO and H_2_S can chemically interact in the tissues to form nitrosothiols which are endowed with their own signaling pathway [51].

**Fig. 1:**
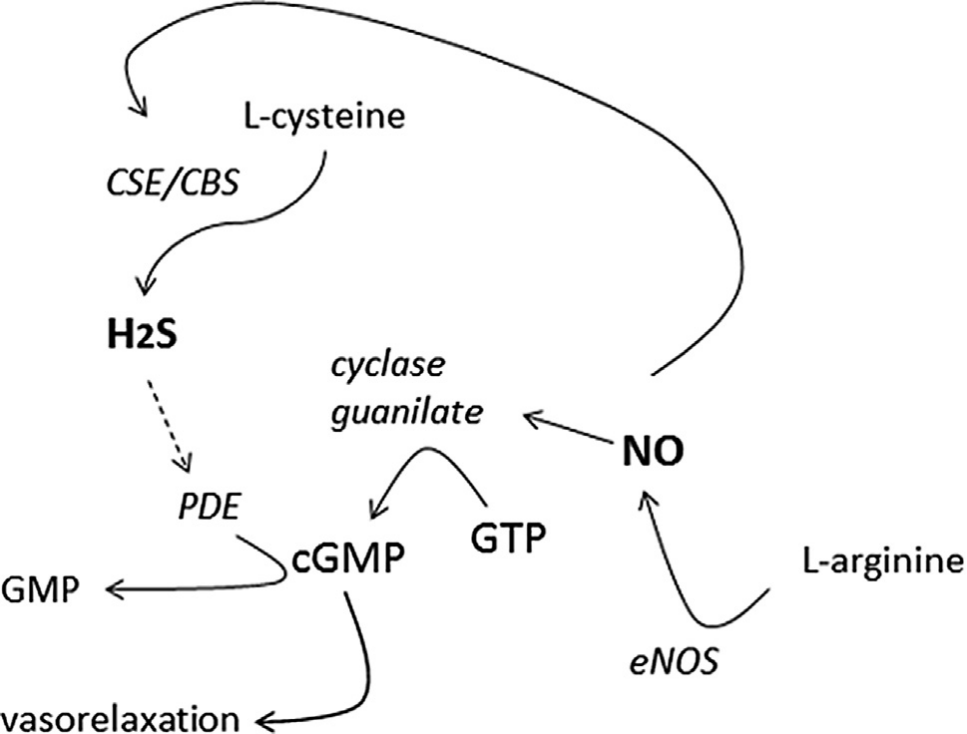
H_2_S increases the cGMP-NO dependent effect by inhibiting the phosphodiesterase-5.

Anyway, such a mutual relationship seems to be missing in the ED condition. In fact, the mutual interplay between NO and H_2_S in rat coronary arteries is attenuated in hypertensive conditions. Interestingly, the ED-related hypertensive status highlights reciprocally independent mechanisms of action of NO and H_2_S, suggesting that cGMP plays a secondary role in meditating H_2_S and NO-related vasoactive responses. Consequently, in this pathological condition, H_2_S plays a compensatory role for the lack of NO-endothelium production, assuring the control of vascular tone [52]. Similarly, in ED-related erectile dysfunction, characterized by a decrease in NO bioavailability, H_2_S compensates the lack of NO [53] (Fig. 1).

On the other hand, other authors reported that NaHS decreases NO formation, reduces eNOS activity and inhibits the uptake of L-arginine in isolated rat aortae and in ECs [54], [55].

Unlike NO, H_2_S modulates the vascular tone not only through a cGMP-dependent manner, but also through cGMP-independent mechanisms. Firstly, it has been demonstrated that H_2_S directly opens ATP-sensitive potassium (K_ATP_) channels in VSMC and causes hyperpolarization which is an event responsible for vasorelaxation [24]. In addition, the involvement of voltage-operated potassium (Kv7) channels has been established in endothelium-denuded rat aortic rings. Similarly to retigabine, which is a Kv7.4 opener, also NaHS activates Kv7.4 currents in Chinese hamster ovary cells (CHO) and produces a significant hyperpolarization [28]. Furthermore, other mechanisms have been proposed, including the inhibition of L-type voltage-operated calcium channels [56].

In several studies, the administration of H_2_S and H_2_S-donors promoted the decrease of blood pressure in hypertensive animals, reversed hypertension-related vascular remodeling and improved the endothelial function in hypertensive humans and rats [13], [24].

Zhao et al., in 2001 observed that intravenous administration of NaHS reduced blood pressure, reversed ED, restored NO bioavailability, limited endothelial ROS production in a rat model of Ang-II-induced hypertension [24], [57]. The reduction of H_2_S plasma levels was also reported in a glucocorticoid-induced hypertension model, typically characterized by ED [38].

The administration of NaHS improves the endothelial function, thanks to the inhibition of oxidative stress and inflammation in spontaneously hypertensive rats. Interestingly, the knocking down of nuclear factor erythroid-2-related factor 2 (Nrf2) abolished such a protective effect [58].

Besides NaHS, polysulfides are considered as natural molecules able to release H_2_S in biological environment. Interestingly, diallyl trisulfide (DATS), diallyl disulfide (DADS) and diallyl sulfide (DAS), mainly occurring in plants of the *Alliaceae* family (for instance garlic), have been demonstrated to be vasorelaxing agents [25] due to their H_2_S-generating property. Indeed, the release of H_2_S is the key event through which polysulfides exert anti-hypertensive effects in L-NAME-treated Wistar rats [59].

In 2008, GYY4137, a widely studied H_2_S slow-releasing compound, has been proven to induce vasodilatation in aortic, renal and cardiac arteries in *in vivo* L-NAME-induced hypertension model. In particular, unlike NaHS that causes immediate, transient falls in blood pressure, GYY4137 (acutely and chronically administered), promoted a slow-developing fall in blood pressure with minimal effects on the heart rate, in rats [60].

More recently, iminothioether chemical group has been recognized as potential new slow H_2_S-releasing moiety endowed with vasorelaxing effects, on aortic and coronary arteries; furthermore, the blood pressure was reduced in L-NAME-treated rats [31].

Interestingly, arylthioamides and, more recently, the natural isothiocyanates occurring in the *Brassicaceae* family, have been recognized as sulfur compounds endowed with H_2_S-releasing properties and with vasoactive effects [29], [30].

Martelli and colleagues firstly demonstrated that erucin, the isothiocyanate deriving from arugula (*Eruca sativa* Mill. belonging to *Brassicaceae* family) is a smart H_2_S-donor, which releases the gasotransmitter only in presence of thiol-groups, or in biological environment. Moreover, erucin showed hyperpolarizing activity on human aortic smooth muscle cells and vasoactive effects in *ex vivo* models; furthermore, in spontaneously hypertensive rats, erucin promoted anti-hypertensive effects [33]. Furthermore, previous studies reported that 4-month oral treatment with sulforaphane (SFN), which is another natural isothiocyanate, reduced blood pressure values in spontaneously hypertensive rats and increased glutathione levels in aortic smooth muscle cells [61], [62].

Noteworthy zofenopril, which is a sulfur-containing ACE inhibitor, showed additional beneficial mechanism unrelated to ACE inhibition, but rather to H_2_S release. Indeed, zofenopril promoted a more marked anti-hypertensive effect than enalapril when administered *in vivo*, and it rescued endothelium-dependent vasorelaxation in *ex vivo* models [63].

Accordingly with the role of H_2_S in the regulation of haemodynamic parameters, the novel triphenyl phosphonium derivatized dithiolethione AP39, a slow H_2_S-donor, significantly lowered systolic blood pressure, decreased heart rate and arterial stiffness in L-NAME treated rats [64].

Taken together these results reveal the crucial role of H_2_S in regulating endothelial function, suggesting that this gasotransmitter, similarly to NO, might be a new tool for treating ED-related hypertension.

## Hydrogen sulfide and atherosclerosis-related endothelial dysfunction

**Fig. 2:**
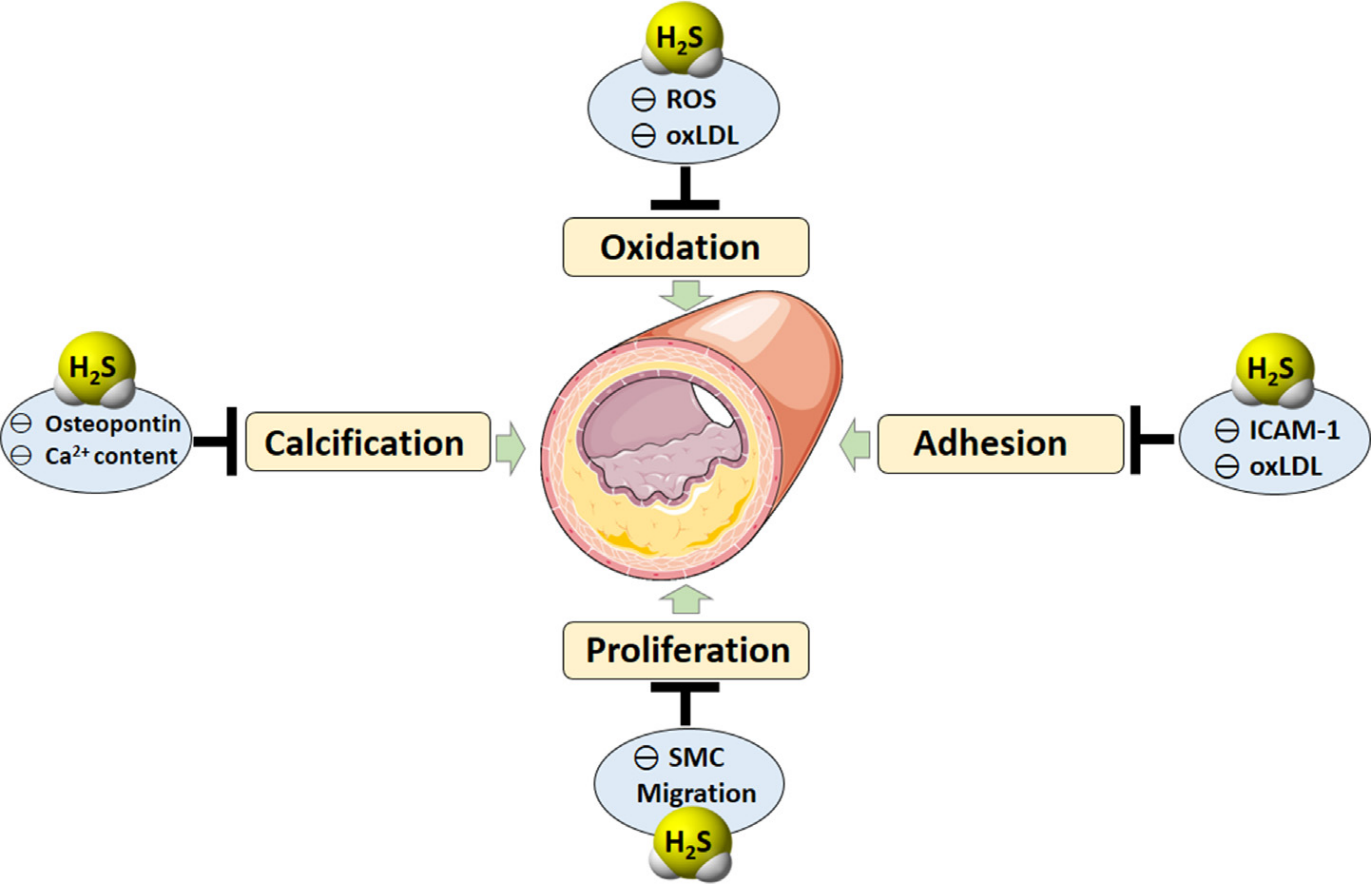
Hydrogen sulfide counteracts the main aspects of atherosclerosis such as oxidation, adhesion, proliferation and calcification by inhibiting: the increase of ROS and ox-LDL, the expression of ICAM-1, the migration of smooth muscle cells (SMC) from the medial to the sub-endothelial layer where they proliferate, incorporate ox-LDL and so contribute to the plaque development; and finally by inhibiting the expression of osteopontin gene and the increase of vessels calcium content and accumulation.

Atherosclerosis is a well-known disease triggered and sustained by vascular inflammation and characterized by intimal deposition of lipoprotein with consequent accumulation of lipids in arterial wall [65]. The rupture of atherosclerotic plaques or formation of thrombi on the atherosclerosis-induced alteration of vascular wall, are the most recognized causes of stroke or myocardial infarction which, in turn, represents the major cause of death among cardiovascular diseases. The onset of atherosclerosis begins as an immune response associated with endothelial dysfunction/disruption due to mechanical lesions, persistent chronic inflammatory stimuli or oxidative damages [66]. ED leads to the deficiency of those mechanisms deputed to maintain vascular homeostasis and prevents ECs inactivation against the pro-atherogenic stimuli, such as the generation of NO, the most known gasotransmitter. Besides the fundamental role of NO at cardiovascular level, in the last decades other two gasotransmitters have been investigated: carbon monoxide (CO) and H_2_S and in particular, the last one was found to derive also from endothelium and to be fundamental in regulating vascular homeostasis [9], [23], [67]. Furthermore, H_2_S preserves the vascular wall integrity, thus preventing the leaking of vascular tree induced by the vascular inflammation. Moreover, it limits the alteration of vascular tissues organization due to the atherosclerosis process [68]. One of the first insights about the role of endogenous H_2_S in atherosclerosis derives from the work of authors who focused the attention on the ability of statins, the main class of drugs used to reduce lipid levels in the blood, to promote the generation of endogenous H_2_S. In particular, these studies reported that the lipophilic atorvastatin and fluvastatin, but not the hydrophilic pravastatin, induced an enhanced H_2_S generation from rats perivascular adipose tissue (by the inhibition of mitochondrial oxidation) and in mice lipopolysaccharide (LPS)-stimulated macrophages (reducing the levels of pro-inflammatory factors such as IL-1β and monocyte chemoattractant protein-1 (MCP-1)) [69], [70]. Besides these observations on the enhanced endogenous H_2_S generation, several studies were carried out to investigate the role of H_2_S in all the main aspects of atherosclerosis such as oxidation, adhesion, proliferation, HHcy and calcification (Fig. 2).

Using pharmacological/experimental tools like the salt NaHS as H_2_S-donor agent, some authors demonstrated that low concentrations of H_2_S were able to directly counteract ROS in vascular smooth muscle and, at the same time, to increase the activity of antioxidant factors and pathways. A very important action of H_2_S is the ability to inhibit the hypochlorite-induced modification of LDL into oxidized LDL (ox-LDL). Ox-LDL represents one of the most potent factors for the onset of atherogenic modifications, because they stimulate the expression of adhesion molecules on ECs triggering the inflammatory process and the atherosclerotic progression [11]. But the role of H_2_S against the adhesion process is promoted not only through the indirect action on ox-LDL: Wang and colleagues demonstrated that H_2_S inhibited the expression of intracellular adhesion molecule-1 (ICAM-1) in TNF-α-stimulated human umbilical vein ECs (HUVEC) and in apolipoprotein-E knockout (apoE^−^/^−^) mice through the inhibition of IkB degradation and NF-kB nuclear translocation. Targeting adhesion molecules is a fundamental strategy to inhibit the link between endothelium and immune cells like monocytes and T cells, since the inhibition of this first step precludes cell migration and the consequent inflammation which sustains atherosclerotic process [71]. However, H_2_S limits the atherosclerotic process also by inhibiting the proliferation of intima and smooth muscle cells. This effect has been demonstrated in rat balloon injured arteries in which NaHS reduced neointimal lesions hyperplasia [72]. Moreover, NaHS also reduced the vascular smooth muscle proliferation, which represents a very harmful phenomenon concurring to the atherosclerotic plaque formation, through the migration of smooth muscle cells from the medial to the sub-endothelial layer where they proliferate, incorporate ox-LDL and thus contribute to the plaque development [73]. Finally, H_2_S seems to also have a role in advanced phases of atherosclerosis, including angiosteosis and calcified vessels which show a down-regulation of CSE expression/activity resulting in a reduced production of H_2_S. Wu and colleagues administered NaHS in a rat vascular calcification model obtained by the administration of vitamin D3 plus nicotine, and observed reduction of calcium content and accumulation in vessels, lower activation of alkaline phosphatase (a calcification inducer) and down-regulation of the expression of osteopontin gene (osteopontin is a glycoprotein involved in bone biomineralization and highly expressed in calcified plaques) [74]. On these bases, the use of H_2_S-donors seems to be a promising strategy to counteract the different phases of atherosclerotic process and several studies were carried out using the salt NaHS as H_2_S-generating molecule. Indeed, the administration of NaHS prevents the development of atherosclerosis in apoE^−^/^−^ high fat-fed mice by downregulating the expression of chemokines receptors CX3CR1 and CX3CL1 both in *ex vivo* explanted plaques and in *in vitro* treated macrophages [71], [75]. Furthermore, Xiong and co-workers in a recent work reported that NaHS enhanced atherosclerotic plaque stability, by increasing cap thickness through the inhibition of VSMCs apoptosis and decrease of the expression of metallopeptidase-9 (MMP-9), an enzyme deputed to collagen degradation, in apoE^−^/^−^ high fat-fed mice [76]. However, on the basis of current knowledge, the H_2_S-donor salts like NaHS, are not the ideal H_2_S-donors because of their property to release H_2_S in a too much rapid way. Thus, the research of novel slow H_2_S-donors and their evaluation on the atherosclerotic process represent a more interesting challenge. Presently, the most investigated slow H_2_S-donor is GYY4137. Its anti-atherosclerotic properties has been investigated both in *in vitro* experiments on murine and human macrophages and in *in vivo* apoE^−^/^−^ high fat-fed mice. In macrophages, GYY4137 inhibited foam cell generation induced by the administration of ox-LDL and down-regulated the expression and the activity of pro-inflammatory/atherogenic factors like NF-kB, ICAM-1 and chemokines. *In vivo,* GYY4137 counteracted the atherosclerotic plaque formation and restored the endothelium-dependent vasorelaxation, also leading to lower ICAM-1, IL-6 and TNF-α expression [77]. A similar investigation on *in vitro* models of macrophages and *in vivo* fat-fed apoE^−^/^−^ mice was carried out also with a particular class of H_2_S-donor, i.e. the H_2_S-donors hybrids [78], [79], [80], [81]. These compounds were synthesized on the basis of previous experience on the NO-donor hybrids, in order to confer the positive effects of the gasotransmitter to selected native molecules. The typical native molecules used in this strategy were well-known drugs, commonly used in clinic, with the aims to improve their pharmacodynamics profile or to correct potential adverse effects of the native compound. This strategy was first applied to non-steroidal anti-inflammatory drugs (NSAIDs) and then applied on many other classes of drugs [82], [83], [84], [85]. As concerns the hybrid H_2_S-donors, the molecule named ACS14 is an early example. It is an H_2_S-releasing aspirin, in which the H_2_S-releasing portion is represented by a dithiolthione moiety. ACS14 was tested both on macrophages stimulated with interferon-γ or LPS and compared with the native molecule aspirin. The results showed that only ACS14, but not aspirin, downregulated CX3CR1 via PPAR-γ-dependent mechanism. The same downregulation of CX3CR1 was also found in brachiocephalic artery of apoE^−^/^−^ high fat-fed mice receiving ACS14 for 12 weeks but not in mice receiving aspirin. This CX3CR1 down-regulation correlated with prevention of atherogenesis and development of atherogenic plaques [86]. Among all the available H_2_S-donors, the natural ones, i.e. H_2_S-donors deriving from plants, represent a very interesting source of H_2_S because of their nutraceutical added value. Garlic polysulfides, such as DADS and DATS, were the first described as natural H_2_S-donors [25]. They have been demonstrated to inhibit the ox-LDL-induced expression of adhesion molecule like vascular adhesion molecule-1 (VCAM-1) and E-selectin. Such a property prevents monocyte adhesion in HUVEC cells and protects eNOS against the damage induced by ox-LDL [87], [88]. In the last years, another family of plants emerged as a source of H_2_S-donor molecules, i.e. the *Brassicaceae* family (Crucifers) in which the secondary metabolites isothiocyanates were well characterized as H_2_S-donors [33], [89], [90], [91]. To date, SFN is the most investigated isothiocyanate and some recent articles described its inhibitory action on vascular inflammation and adhesion molecule expression in advanced glycation end products (AGEs)-treated HUVEC-cells and in AGEs-perfused rat aortas [92]. Moreover, in a more specific article, SFN was administered to high fat-fed rabbits and the results showed that SFN reduced the intima/media ratio, the levels of total cholesterol, LDL, C-reactive protein, lactate dehydrogenase and the NF-kB expression. At the same time, SFN increased high-density lipoproteins (HDL), reduced glutathione levels and improved the aortic endothelium-dependent vasorelaxing effect after administration of acetylcholine, demonstrating an impressive impact on several atherogenic factors [93]. Finally, a very recent and interesting work by Bibli and colleagues investigated the link between H_2_S, ED and atherosclerosis, purposing an original mechanism of action. In particular, they observed that both in cultured ECs and in mice, endogenous CSE-derived H_2_S induced sulfhydration and dimerization of the RNA-binding protein human antigen R (HuR). Conversely, such a HuR sulfhydration was inhibited in atherosclerosis, leading to increased expression of CD62 antigen-like family member E (CD62E) and cathepsin S mRNAs, which are related to endothelium activation and atherogenic stimuli. As a further confirmation, the administration of the polysulfide SG1002, a slow polysulfide donor, in ECs isolated from CSE knockout mice, restored HuR sulfhydration with consequent decrease in levels of CD62E protein and in monocyte adherence. Moreover, SG1002, administered to apoE^−^/^−^ CSE knockout mice submitted to partial carotid ligation, limited the plaque formation and the HuR link to cathepsin S mRNA, demonstrating an H_2_S-induced anti-atherogenic effect [94].

## Hydrogen sulfide and diabetes-related endothelial dysfunction

It was well established that ED could lead to a series of pathologies including cardiovascular diseases, atherosclerosis, and hypertension and is closely related to diabetes. Indeed, the alteration in endothelial functionality is extremely evident in patients affected by diabetes that is a pathological condition characterized by a high risk of cardiovascular disease, ascribed to the severe adverse effects of oxidative stress and hyperglycemia. Also, prediabetic patients, characterized by impaired glucose tolerance and impaired fasting glucose, develop with more frequency important cardiovascular disease [95]. These findings largely suggest that anomalies in carbohydrate metabolism are responsible for a progressive degeneration of healthy cardiovascular functions, where ED represents the first step of the undesirable events leading to a variety of pathological conditions [96]. ED in diabetes is linked to reduced bioavailability of NO, slightly compensated by endothelium-dependent hyperpolarization and/or production of prostacyclin, and increased production or action of endothelium-derived vasoconstrictors. This condition also leads to microvascular complications, including nephropathy, neuropathy and retinopathy, primarily characterized by abnormal angiogenesis, enhanced oxidative stress, increased production of inflammatory factors, decreased release of NO and impaired endothelial repair.

Additionally, the generation of AGEs is particularly intensified due to presence of elevated concentration of glucose, contributing to vascular complications.

**Fig. 3:**
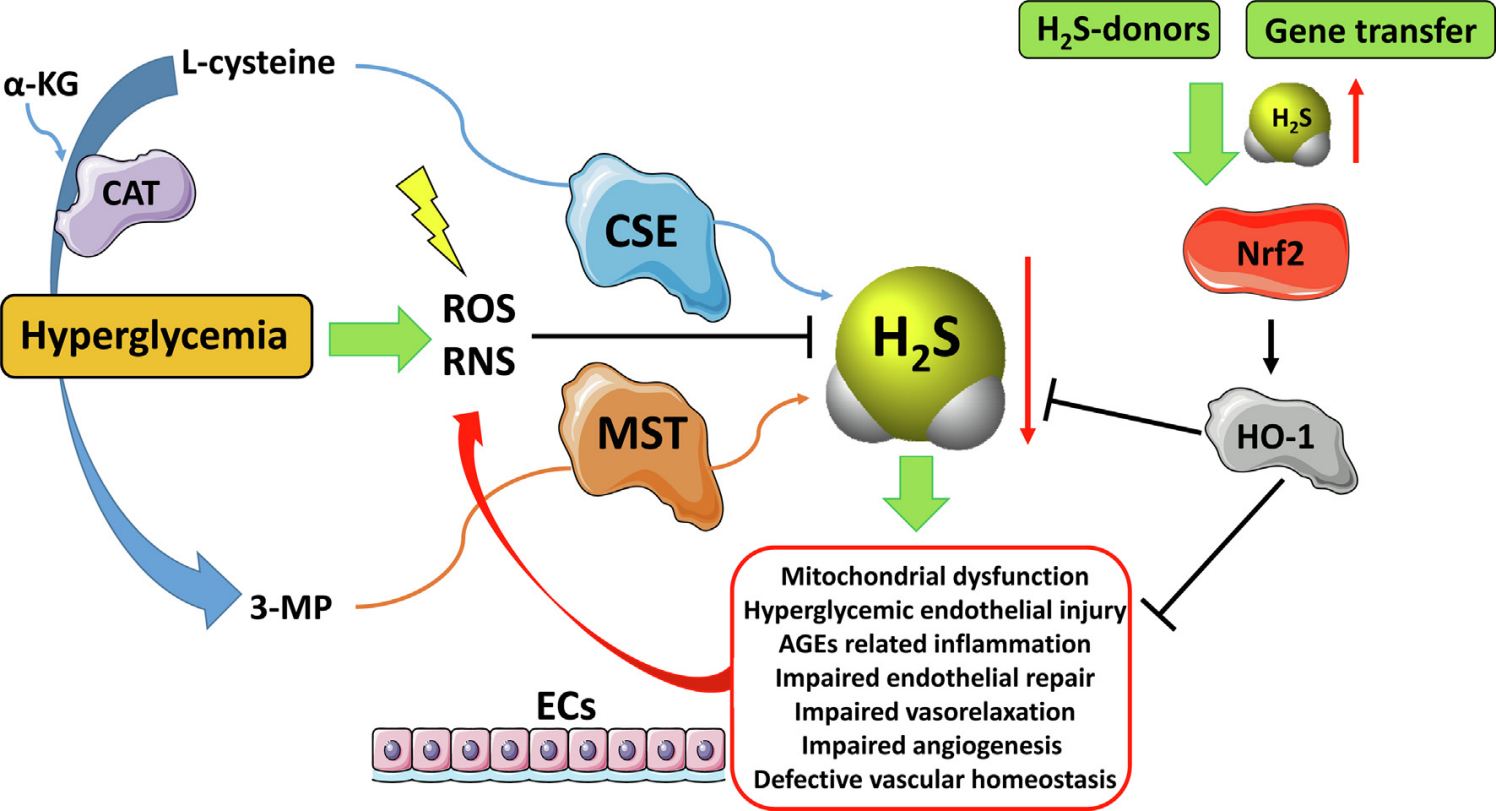
Schematic representation of the effects of hyperglycemia on ECs. This event caused ED due to the formation ROS/RNS, leading to a decrease of gasotransmitter H_2_S. H_2_S deficiency is implied in a series of alteration of cellular processes highlighted in a red line box. The exogenous H_2_S supplementation counteracts these dysfunctions stimulating the expression of HO-1 in an Nrf2-dependent manner. The picture was prepared by using Smart SERVIER MEDICAL ART provided under Creative Commons Attribution 3.0 Unported License. ROS/RNS (Reactive Oxygen Species/Reactive Nitrogen Species); α-KT (α-ketoglutarate); CAT (cysteine aminotransferase); CSE (cystathionine γ-lyase); MST (3-mercapto sulfotransferase); 3-MP (3-mercaptopyruvate); ECs (endothelial cells); Nrf2 (nuclear factor erythroid 2-related factor 2); HO-1 (heme oxygenase 1); AGEs (advanced glycation end products). (For interpretation of the references to color in this figure legend, the reader is referred to the web version of this article.)

In diabetic patients, ED seems to be a consistent hallmark which leads to an alteration of gasotransmitters i.e. NO, CO and H_2_S production [10], [97]. In fact, H_2_S is mainly produced in ECs and is involved in the fine regulation of endothelial integrity and functions. For this reason, altered H_2_S bioavailability has been proposed as a novel marker of ED advancement and prognosis [10]. According to the mentioned observations, an initial dysfunction of ECs triggers metabolic and vascular modifications associated with type 2 diabetes (Fig. 3).

H_2_S exhibits positive effects on vascular endothelium, emerging as a central regulator of vascular disease in diabetes, due to its crucial role in maintaining healthy functions in ECs [98], [99]. In addition, H_2_S contributes to wound healing and VSMC relaxation, thus decreasing blood pressure and platelet aggregation [100], [101]. It also exerts potent anti-apoptotic, antioxidant, angiogenic responses and anti-inflammatory. H_2_S exerts several of its pharmacological effects by targeting proteins through S-sulfhydration. In this reaction, an atom of sulfur is added to the thiol groups of reactive cysteine residues resulting in the formation of hydropersulfides [102]. More recently, H_2_S has been demonstrated to limit ED, nephropathy, cardiomyopathy, retinopathy in experimental animal models of diabetes [103], [104], [105], [106], underlining the protective nature of this molecule [107]. Reliably, a shortfall in H_2_S homeostasis is involved in the pathogenesis of hyperglycemic endothelial injury. Consequently, the use of H_2_S-releasing compounds or gene therapy to increase the level of endogenous H_2_S could help in restoring endothelial function and antagonizing the progression of related diseases. For these reasons, we discuss about the potential role of H_2_S-releasing agents as a novel therapeutic option for the treatment of diabetes-related ED.

In a ground-breaking study, Xie and colleagues [108] highlighted the significant role of H_2_S in diabetes. They demonstrated, that this gasotransmitter counteracts diabetes-accelerated atherosclerosis, in a model of streptozotocin-induced diabetic high fat-fed mice. In the mentioned study they demonstrated that chronic administration of GYY4137, a slow-releasing H_2_S-donor, reduced atherosclerotic lesion size in atheroprone animals, independently by any change in circulating blood cholesterol and glucose. Xie and colleagues also observed that the anti-atherosclerotic effect of GYY4137 is linked to a reduction in the amount of macrophages within the plaque, in the expression of the adhesion receptors and the production of superoxide. Similar effects also emerged when peritoneal macrophages or ECs are treated with ox-LDL and high concentrations of glucose, which is a condition that mimics the *in vivo* environment encountered in diabetes. In addition, the researchers observed that GYY4137 is able to activate the transcription of Nrf2 through the specific S-sulfhydration of amino acidic residue cysteine 151 (Cys151) in Kelch-like ECH-associated protein 1 (Keap1). Keap1 decrease the activity of Nrf2 by promoting its degradation through the ubiquitin–proteasome pathway. Fascinatingly, in diabetic mice the antiatherogenic action of GYY4137 is completely abolished when Nrf2 is deleted or silenced, highlighting that the action is mediated by Nrf2. Moreover, the compound increases the trascription of HO-1 in an Nrf2-dependent manner, and inhibition or depletion of HO-1 completely reduces the beneficial actions of GYY4137, indicating that HO-1 is involved in the antiatherogenic effects of H_2_S. Notably, Xie and coworkers detected significantly lower levels of plasma H_2_S in diabetic mice, and demonstrated that the administration of GYY4137 restored physiological levels of H_2_S. In patients with diabetes and other diabetic animal models a reduction in circulating H_2_S has also been reported [109], [110], confirming an H_2_S deficiency in diabetic pathology. The cause for this decline is still unclear but may be associated to alterations in the global activity of H_2_S-generating enzymes, the microbial reduction of sulfate in the intestine, the generation of H_2_S from other sources and/or the metabolism of H_2_S in diabetes. Clearly, more detailed studies are needed to solve this issue and to discover optimal circulating concentrations of H_2_S needed to maintain vascular homeostasis in diabetes.

Gero and coworkers [111] studied the therapeutic potential of two novel mitochondria-targeted hydroxythiobenzamide and anethole dithiolethione H_2_S-donors (AP123 and AP39 respectively) in diabetic cardiovascular complications, which are, at least in part, promoted by mitochondrial ROS production in ECs. High concentration of glucose in the bloodstream is responsible for superoxide production in the mitochondria and triggers modifications in the mitochondrial membrane potential, leading to mitochondrial dysfunction. Since H_2_S supplementation reduces the mitochondrial oxidant production and shows efficacy against diabetic vascular damage *in vivo*, the role of AP39 and AP123 has been evaluated in preventing hyperglycemia-induced oxidative stress and metabolic alterations in microvascular ECs. AP39 and AP123 reduced hyperglycemia-induced mitochondrial membrane hyperpolarization and repressed the mitochondrial ROS production. Both H_2_S-releasing compounds improved the cellular metabolism and enlarged the electron transport at respiratory complex III. The high potency and long-lasting effect provoked by these H_2_S-donors suggest that these classes of compounds could be useful for the treatment of diabetic vascular complications.

Another work by Ng and colleagues [112] highlighted how NaHS chronic treatment is able to decrease the oxidative stress and exerts vasoprotective effects, improving endothelial function in streptozotocin-induced diabetes C57BL6/J mice. In particular, NaHS treatment restored endothelial function reverting diabetes-induced vascular dysfunction by improving NO efficacy and reducing superoxide production in the mouse aorta.

In another study, Ma and colleagues [113] demonstrated that the exogenous supplementation of H_2_S ameliorated diabetes-associated cognitive decline (DACD). This disorder is related to ED and represents one of the complications of diabetes often leading to cognitive impairment and an augmented risk of dementia. The researchers investigated the anti-inflammatory and anti-apoptotic effects of H_2_S on DACD and demonstrated that H_2_S improved the spatial learning and memory abilities of the diabetic mice by modulating the IL-23/IL-17 axis and the mitochondrial apoptotic pathway, which were discovered to be associated with DACD. Accordingly, H_2_S treatment may help preventing the progression of apoptotic hippocampal neurons and EC in diabetic mice and could represent an innovative therapeutic strategy for the treatment of DACD.

Recently, H_2_S-donors derived from natural source are attracting the attention of researchers for the discovery of novel therapeutics strategies to treat different diseases including diabetes and diabetes-related ED. In particular, organosulfur compounds (OSCs) occurring in plant extracts can behave as cardio protective agents in type 2 diabetes, due to their immunomodulatory, antioxidant, anti-inflammatory and hypoglycemic effects. OSCs contained in garlic (*Allium* sp.), due to their properties, seem to be a valuable dietary support in type 2 diabetes, as reported in several studies. Garlic OSCs are able to produce H_2_S, and several their beneficial effects are probably due to this property. In fact, recently, many studies reported the relevant effects of exogenous and endogenous H_2_S in diabetes, including *in vivo* and *in vitro* experiments and clinical trials as nicely reviewed by Melino and colleagues [114]. Briefly, garlic has been demonstrated to exert beneficial effects in diabetes, including hypocholesterolemia, hyperinsulinemia, anti-lipid peroxidation, hypoglycemia, hypotriglyceridemia and anti-glycation [114]. As reported by Padiya and colleagues [115], garlic extract improved insulin sensitivity in a model of insulin-resistance, associated with metabolic syndromes in fructose-fed diabetic rats treated with garlic homogenate. In particular, they showed a significant decrease in serum glucose, triglycerides and uric acid levels. Garlic also normalized the serum levels of endogenous NO and H_2_S after fructose feeding, suggesting also an improvement in the endothelium functionality and an attenuated metabolic syndrome.

More recently, Thomson and coworkers showed the antidiabetic and antioxidant potential of aged garlic extract in streptozotocin-induced diabetic rats. The diabetic rats showed an elevated blood glucose, serum cholesterol and triglycerides, erythrocyte glycated hemoglobin and kidney and liver lipid peroxidation. Interestingly, the treatment with aged garlic extract positively reverted the diabetic alterations to significantly lower levels than those measured in diabetic rats.

Baluchnejadmojarad and colleagues [116] investigated the antidiabetic, antioxidant, anti-inflammatory and normotropic property of S-allyl cysteine (SAC), the most abundant organosulfur bioactive molecule in aged garlic extract. The mentioned work was designed to assess the therapeutic potential of SAC in limiting memory and learning deficits in streptozotocin-induced diabetic rats and to evaluate the involvement of toll-like receptor 4 (TLR4), Nrf2, nuclear factor-kappa B (NF-κB), and HO-1 signaling cascades. SAC treated rats showed a decrease of serum glucose, and an improvement in spatial recognition memory in several *in vivo* tests. Moreover, SAC reduced lipid peroxidation marker malondialdehyde (MDA), acetylcholinesterase activity, and augmented antioxidant defensive system including catalase, superoxide dismutase (SOD), and reduced glutathione in hippocampal lysate. In addition, SAC lowered hippocampal TLR4, NF-kB and TNF-α and prevented reduction of HO-1 and Nrf2 in diabetic rats. Taken together, chronic SAC treatment ameliorated cognitive deficits in diabetic rats. Unfortunately, these encouraging results were not found during a recent clinical trial.

Also, sulfur-containing compounds deriving from the *Brassicaceae* family could represent valuable starting point for drug development against diabetes and its related diseases. In fact, SFN, an isothiocyanate found in Broccoli, has been proposed as potential antidiabetic agents. In a clinical study SFN was administered (112 or 225 μmol/day for 4 weeks) to diabetic patients. The results showed reduction in fasting glucose, inflammatory markers and serum insulin. Moreover, in the same randomized double-blind placebo-controlled clinical trial, a decrease in serum triglycerides was observed. Furthermore, they reported a lowered density of lipoprotein ox-LDL/LDL ratio, reduced atherogenic index of the plasma and an increase in HDL concentration in patients receiving the highest dose of SFN [117], [118]. Axelsson and colleagues observed that SFN reduced hepatic glucose production and improved glucose control in patients with type 2 diabetes. Patients were treated with broccoli sprout extract containing SFN which interestingly, reduced glycated hemoglobin (HbA1c) and fasting blood glucose in obese patients with dysregulated type 2 diabetes [119]. Moreover, SFN was shown to arrest the progression of type 1 diabetes in streptozotocin treated mice by inhibiting oxidative stress-induced beta cell damage and protecting ECs against diabetes-induced complications [120]. SFN is also known to prevent aortic damage in streptozotocin-induced diabetes in type 1 diabetes mice [121].

Additionally, many authors demonstrated that inhibition of AGEs formation may be a valid therapeutic strategy to improve vascular complications in diabetes. AGEs directly blocks NO activity and produces ROS in vascular endothelium. The diabetic condition is characterized by low levels of NO at the vascular level due to oxidative stress and increased AGEs levels which contribute to the worsening of the endothelium functionality. Diabetic rats treated with SFN showed a significant decreased in vascular oxidative stress, and concomitantly increased NO bioavailability, partially explaining the beneficial effects on vascular function [122].

Summarizing, in diabetes, H_2_S-donors showed a wide spectrum of beneficial effects counteracting diabetic complications. The evidence on the beneficial effects of H_2_S-donors included the restoration of H_2_S level, since in diabetes there is a decrease of H_2_S bioavailability [123]. In addition, in overweight and obese patients, the H_2_S levels in plasma are reduced, and this event represents a feature of metabolic syndrome and it is commonly observed in type 2 diabetes [40]. H_2_S or its donors have an antiatherogenic property and act by inhibiting the oxidation of LDL as a result of scavenging the free radicals (notably hypochlorous acid and hydrogen peroxide), inhibition of the myeloperoxidase enzyme, and inhibition of the foam cell formation by several mechanisms [124]. Based on this evidence, the use of H_2_S-releasing compounds is a highly feasible approach that can be used to augment H_2_S levels in diabetic patients. Along with inorganic salts and natural H_2_S-donors, many synthetic compounds have been developed, possessing better H_2_S release kinetics and pharmacokinetic profiles [91]. Alternatively, endogenous circulating levels of H_2_S may be increased by gene delivery of H_2_S-generating enzymes or by the supplementation of dietary sulfur that is readily converted to H_2_S by the gut microbiome. Strategies employing these approaches in clinical study, could provide in the near future innovative drug candidates for the prevention and treatment of diabetes and its related diseases.

## Hydrogen sulfide and hyperhomocysteinemia-related endothelial dysfunction

**Fig. 4:**
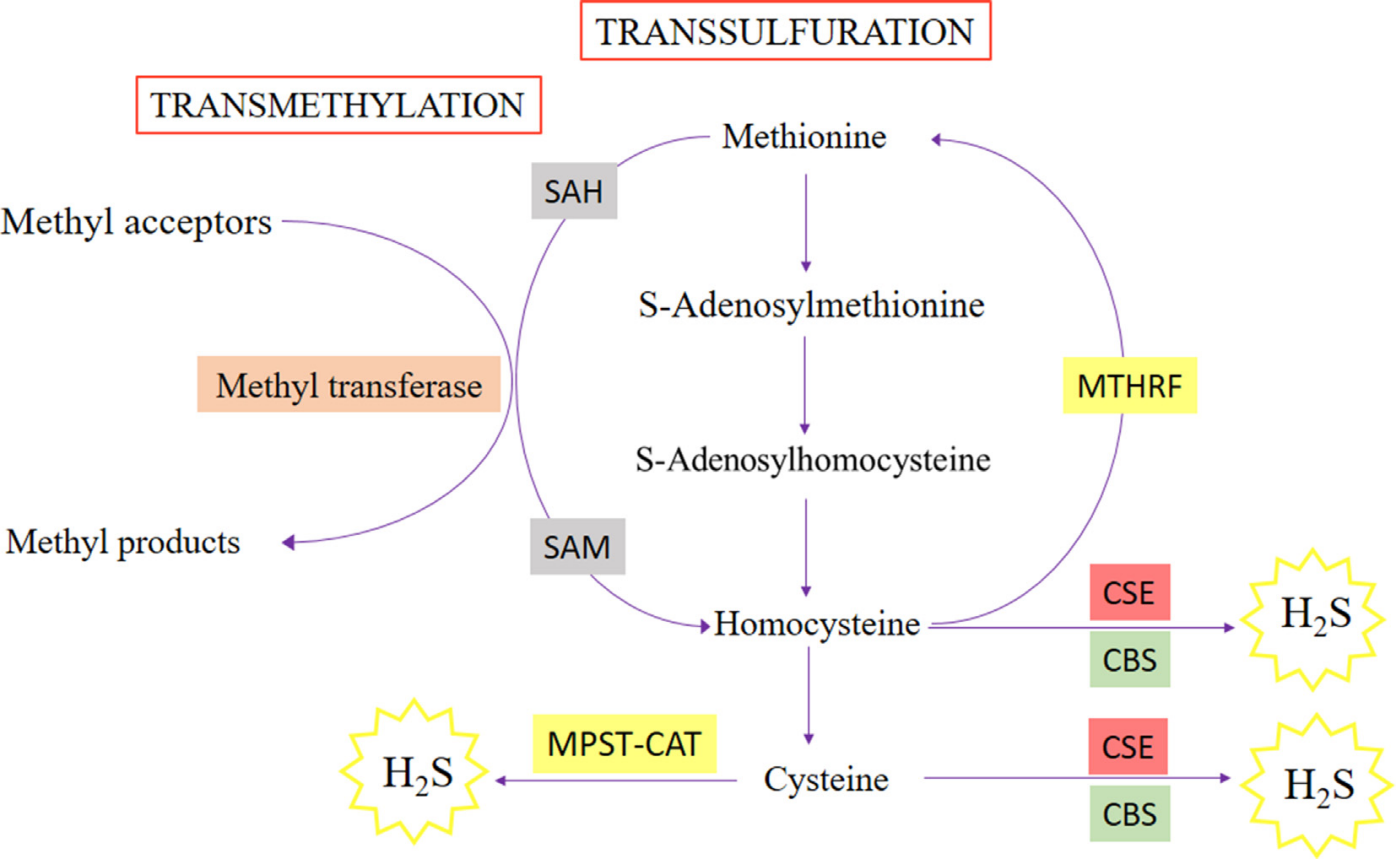
Homocysteine (Hcy) metabolism in physiological conditions. Hcy derives from the hydrolysis of S-Adenosyl-L-homocysteine (SAH) and is metabolized through *trans*-sulfuration pathway and remethylated to methionine via the remethylation pathway. When methionine is in excess, Hcy goes to *trans*-sulfuration pathway which is catalyzed by cystathionine β-synthase (CBS) and cystathionine γ-lyase (CSE).

Hcy is a thiol-containing amino acid deriving from essential amino acid methionine through remethylation reaction, mainly occurring in the liver. Hcy metabolism, described more in detail in Fig. 4, involves three different enzymes, such as betaine-Hcy methyltransferase (BHMT), CBS and CSE [125].

About the 5–10% of the total daily cellular production of Hcy is not metabolized within the cells but is released into the bloodstream compartment. In healthy subjects the concentration of free Hcy is maintained below 15 μmol/L thanks to a constant renal clearance [126]. If the regulation of intracellular Hcy levels is disrupted, Hcy extrusion to the plasma compartment increases and consequently high levels of Hcy are accumulated in the bloodstream [127]. The free Hcy excess into the blood prevents cell toxicity, but on the other hand, exposes vascular tissue to a possible deleterious effect [128].

High tissue and plasma Hcy levels (i.e. HHcy) is an independent risk factor for cerebrovascular, cardiovascular and peripheral artery disease [129]. The pathogenesis of HHcy is mainly due to impaired renal clearance, malnutrition that may cause vitamin B12 and folate deficiency, methionine-rich meat diet or polymorphism in CBS gene [125]. HHcy-related vascular diseases are strictly linked to ED since elevated levels of Hcy have been reported to be directly harmful for the ECs both in *in vitro* and *in vivo* models. Consequently, Hcy exerts its adverse effects by disturbing endothelial function [130]. Accordingly, many studies demonstrate a clear association between HHcy and coronary artery disease, atherosclerosis, hypertension and neurodegenerative diseases [131]. Indeed, HHcy can be considered as a predictive biomarker of cardiovascular mortality in patients affected by coronary artery disease, and plasma levels of Hcy correlate with the severity of atherosclerosis [132].

The HHcy-related ED is a complex mechanism that triggers secondary cardiovascular diseases including atherosclerosis, hypertension and neurodegeneration by inducing oxidative stress and inflammatory status. HHcy increases ROS production through different mechanism, as Hcy autoxidation. Indeed, Hcy can bind plasma proteins or a second Hcy molecule through a disulfide bridge. In these cases, the free thiol group of Hcy undergoes to autoxidation, generating H_2_O_2_, ROS, hydroxyl radical and superoxide anion which can in turn react with NO to produce peroxynitrite, leading to a decrease in the bioavailability of NO [133]. Secondary, HHcy creates a disequilibrium between antioxidant and oxidant enzymes, i.e. inhibition of SOD or activation of NADPH oxidases [134]. Furthermore, both *in vitro* and *in vivo* experiments have reported that HHcy can block the glutathione peroxidase activity and HO-1, contributing to ROS accumulation and exacerbating the ECs damage [135], [136].

**Fig. 5:**
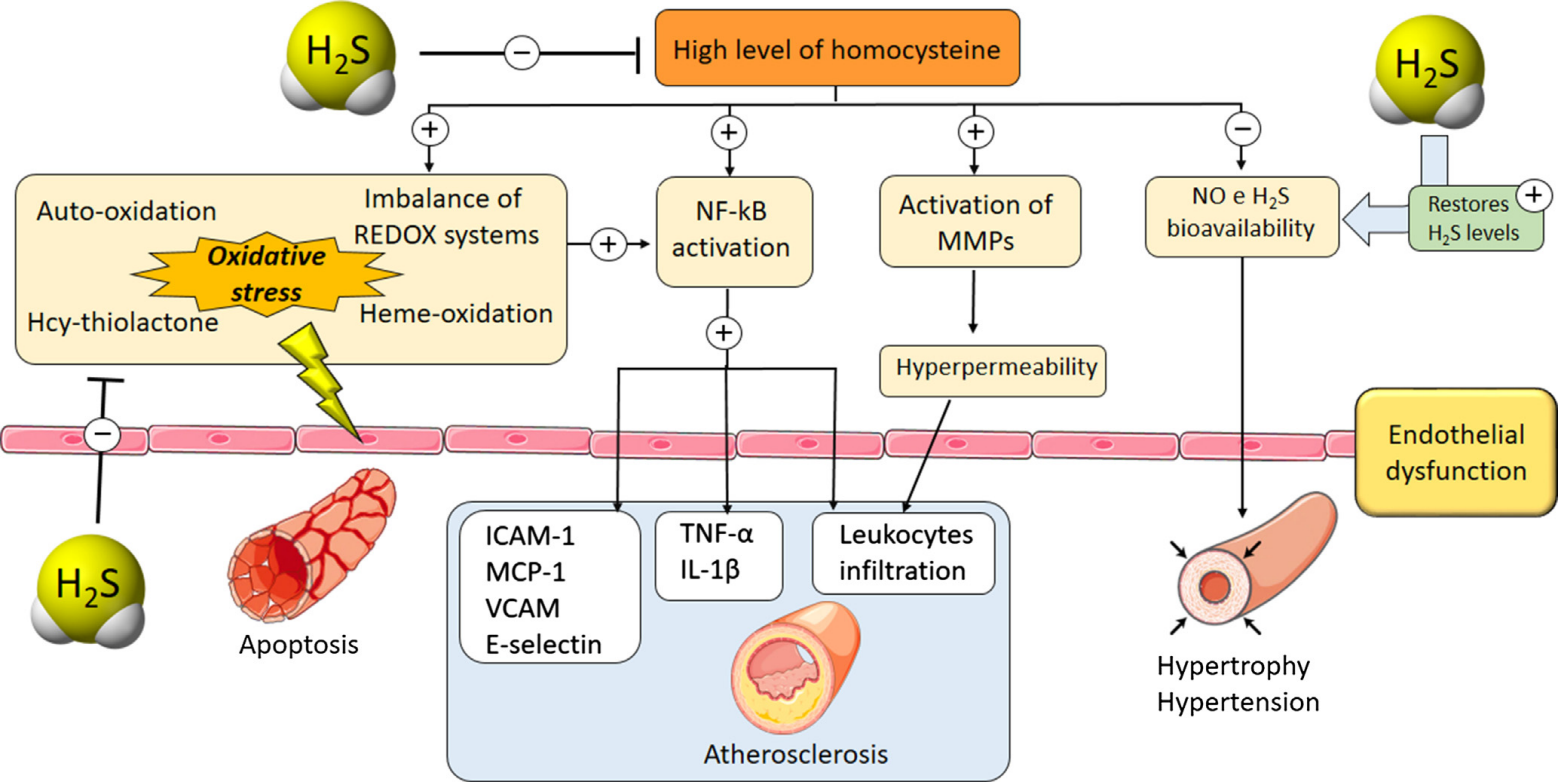
Hyperhomocysteinemia (HHcy) condition is responsible for the activation of several signaling pathways which lead to harmful conditions for endothelium functionality. Firstly, HHcy creates a severe oxidative stress due to the formation of reactive intermediates (Hcy-thiolactone, Hcy persulfide etc.) which contribute to the imbalance of crucial REDOX enzyme systems (inhibition of SOD or activation of NADPH oxidases). Furthermore, HHcy and ROS activate NF-kB signaling pathway resulting in a marked inflammatory response through the production of cytokines (TNF-α, Il-1β); simultaneously pro-atherosclerotic mediators are overexpressed (ICAM-1, MCP-1, VCAM, E-selectin) contributing to the formation of atherosclerotic lesion. HHcy also activates MMPs increasing endothelium permeability which facilitates leukocyte infiltration in the underlying tissues. Furthermore, HHcy is responsible for a decrease in the bioavailability of fundamental vasodilator molecules, including NO and H_2_S promoting vascular hypertrophy and hypertension. In this context, H_2_S is able to beneficially act at different levels: H_2_S is a well-known ROS scavenger, thus exogenous administration of H_2_S-donor significantly reduces the oxidative stress, limiting the vascular inflammation. Furthermore, H_2_S directly reduces the Hcy plasma levels, thus preventing the activation of the downstream pathways. Finally, H_2_S limits the HHcy-induced hypertension and vascular hypertrophy.

Beside the dramatic increase of the oxidative stress, HHcy also promotes a proinflammatory effect, through the activation of those genes that regulate the expression of chemokines, cytokines and adhesion molecules, such as NF-κB: Hcy and ROS promote NF-κB translocation into the nucleus where activates specific target genes. In particular, HHcy-related NF-κB activation has been demonstrated to promote the expression of ICAM-1, MCP-1, VCAM-1 and E-selectin [137]. HHcy induces vascular inflammation also involving migration of leukocytes from the vessels to the underlying tissues by increasing the expression of CD11B/CD18 proteins which are responsible to promote the interaction between the endothelium and the inflammatory cells [138]. Moreover, HHcy causes vascular fibrosis due to activation of matrix metalloproteinases (MMPs), leading to the alteration of extracellular matrix (ECM) metabolism and promotion of collagen deposition [139] (see Fig. 5).

The functional importance of the endothelium also lies in the maintenance of antithrombotic surface for blood flow and hemostasis by controlling the equilibrium between the production of pro- and anti-coagulant molecules. HHcy promotes the activation of coagulation process by inhibiting thrombomodulin-dependent protein C activation, impairing production of von Willebrand factor (VWF) and activating factor V [140]. Consistently, high levels of Hcy have been reported in patients affected by acute coronary artery disease and venous thrombosis [141].

HHcy is also involved in atherosclerosis disease. Indeed, the apoptotic process occurring in ECs is an important hallmark of atherosclerosis and leads to the generation and the disruption of atherosclerotic plaque. In this process, HHcy mediates apoptotic cell death of smooth muscle and ECs [142]. Other studies demonstrated the contribution of HHcy-induced inflammation in the atherosclerotic plaques using hyperhomocysteinemic apoE^−^/^−^-deficient mice, revealing the activation of NF-κB and downstream proinflammatory mediators in atherosclerotic lesions [143]. Moreover, Hcy high plasma levels induced ECs apoptosis due to endoplasmic reticulum stress, enhancement in ROS production and Hcy-thiolactone generation [144].

The clear association between cardiovascular disease and HHcy contributed to highlighting the great importance of ECs and demonstrated the interference of Hcy in maintaining the endothelium functionality. In particular, HHcy reduces NO bioavailability [145], interferes with the H_2_S signaling pathway [146], enhances oxidative stress [147], perturbs lipoprotein metabolism and protein N-homocysteinylation [148].

Among the vasodilators endothelial factor, H_2_S emerged as an important gasotransmitter regulating endothelium functionality and cardiovascular homeostasis and many evidences demonstrate that HHcy leads to severe deficiency in H_2_S bioavailability. Accordingly, exogenous supplement of H_2_S is likely to be considered a pharmacological tool for limiting the HHcy-related ED.

In particular, impaired H_2_S bioavailability has been related to prognosis and progression of HHcy-related ED, since high levels of Hcy has been demonstrated to suppress expression/activity of the H_2_S-generating enzymes CBS and CSE, reducing the endogenous biosynthesis of H_2_S in cultured ECs [149]. The impairment of H_2_S production linked to high level of Hcy has been demonstrated by Man-Hong and colleagues who showed that intracerebroventricular injection of Hcy caused ER stress in the cerebral ECs, worsened the learning and memory, and downregulated CBS expression resulting in a limited generation of H_2_S. Since H_2_S plays an important role in regulating ER stress, this study suggested that Hcy-induced loss of learning and memory is related to the lowered bioavailability of endogenous H_2_S and the increase in ER stress [150]. Indeed, the restoration of H_2_S levels with NaHS treatment significantly limited cerebrovascular neurodegeneration and dysfunction. Such a protection is associated with the reduction of oxidative stress, associated with decreased of malondialdehyde and superoxide, and with the simultaneous increased of glutathione, SOD, catalase [151], [152]. The above-mentioned studies demonstrated that H_2_S supplement with the administration of NaHS increases the levels of antioxidant molecules. This is probably due to the H_2_S ability in enhancing the activity of γ-glutamylcysteine synthetase and upregulating transport of cysteine, strictly related to the synthesis of glutathione [153]. Furthermore, H_2_S may also behave like a scavenger towards the HHcy-induced hemoglobin oxidation species, limiting the lipid peroxidation in vessel endothelium [154].

Different *in vitro* cell culture experiments showed that the treatment with NaHS significantly reduced RNS/ROS levels and normalized redox enzyme levels in VSMC and bEnd3 ECs (brain ECs) pretreated with methionine, which is the precursor of Hcy [155], [156]. Further investigations highlighted the crucial role of mitochondria in Hcy-induced RNS/ROS production leading to mitochondria toxicity and cell death through the activation of N-methyl-D-aspartate-R1 (NMDA-R1) in ECs. Moreover, Hcy treatment lead to an impairment in ATP mitochondrial production. The treatment with NaHS restored the ATP levels and prevented the NMDA-R1 activation, thus protecting ECs from Hcy damage [157]. The authors also demonstrated that the epigenetical modulation of CSE expression enhanced the beneficial role of endogenous H_2_S in inhibiting HHcy-mitochondrial toxicity and reducing mitochondrial superoxide formation [157]. In addition, Hcy enhanced ROS production in isolated mitochondria from mouse aortic ECs, triggering the mitophagy process. Interestingly, epigenetical modulation of H_2_S generating enzymes (i.e. CSE or CBS), limited mitophagy by inhibiting ROS production [156].

Another study reported that dietary supplementation with methionine in drinking water promoted the increase of Hcy and inflammatory cytokines (TNF-α, IL-1β) plasma levels, associated with impairment of endogenous H_2_S production in C57BL/6 mice. Moreover, HHcy significantly downregulated CSE expression in the intestinal macrophages extracted from the HHcy-mice compared to control mice. To clarify whether H_2_S impairment was correlated with the increase of pro-inflammatory cytokines, methionine-fed mice were treated with GYY4137, an H_2_S-releasing molecule, and DL-propargylglycine or D-penicillamine, which are two CSE inhibitors: GYY4137 markedly decreased TNF-α and IL-1β amount in the plasma of methionine-treated mice; on the contrary, DL-propargylglycine and D-penicillamine exacerbated the increase of these cytokines level [158], [159].

In addition, Hcy sustains and promotes ED and causes vascular hyperpermeability by increasing the activity of MMPs which are clearly involved in the atherosclerotic plaque formation, since they can facilitate vascular remodeling by digesting the matrix detaching the intima from the media tunica and allow smooth-muscle cell migration from the media to the intima. H_2_S is able to inhibit Hcy-induced MMPs activity leading to a decrease of smooth muscle hyperproliferation and suppression of vascular remodeling and inflammation [160], [161].

Beside the downregulation of the H_2_S generating enzymes, Hcy can be also considered as a scavenger of hydrosulfide anion to form Hcy persulfide. In HHcy conditions, the accumulation of Hcy persulfide weakened the cardioprotective effect of NaHS in HHcy animals subjected to ischemia–reperfusion injury [162].

HHcy has been demonstrated to reduce NO biosynthesis by inhibiting eNOS and activating arginase enzyme. *In vitro* and *in vivo* studies reported that H_2_S is able to counteract such a NO decrease by mainly increasing eNOS mRNA synthesis, maintaining soluble guanylate cyclase and inhibiting PDE-5 activity [26], [52].

Although the promising results about the and effectiveness of NaHS in reducing the HHcy-related ED, this H_2_S-donor is characterized by a fast H_2_S donation which makes it unusable in the clinical practice due to its toxicity and short half-life. Consequently, the research in this field focused on developing safe H_2_S-based drugs, with reduced toxicity and enhanced efficacy, in particular the H_2_S prodrug SG1002 is likely to be a very promising pharmacological therapy for heart failure. SG1002 is an inorganic mixture (sodium polysulthionate) containing S_8_, Na_2_SO_4_, Na_2_S_2_O_3_, Na_2_S_3_O_6_, Na_2_S_4_O_6_, and Na_2_S_5_O_6_ and promoted cardiac remodeling and afterload in the CBS+/- hyperhomocysteinemic mouse model. CBS+/− mice developed several symptoms of early and late cardiac modification, including hypertrophy and fibrosis that lead in enhanced afterload on the heart. These tissue modifications were limited by dietary supply of the SG1002 which normalized the histological and molecular measurements of hypertrophy and fibrosis, since SG1002 significantly decreased collagen accumulation in the left ventricle of hyperhomocysteinemic mice, inactivated MMP and limited cardiomyocyte size [163].

Also naturally occurring isothiocyanates may be exploited for the treatment of HHcy-related ED, since many studies demonstrated their ability to donate H_2_S [89]. Indeed, preliminary data reported that SFN, which is a natural isothiocyanate able widely present in the *Brassicaceae* family, exerts protective effects on Hcy-induced oxidative stress *in vitro* by reducing ER stress and behaving as ROS scavenger [164].

## Conclusions

The ED refers to as the loss of ECs physiological functions caused by different risk factors, including hyperglycemia, hypercholesterolemia and HHcy. ECs respond to such pathological conditions by enhancing the oxidative stress along the vascular tree, leading to several damaging events resulting in the inability of the endothelium to exert its fundamental activities, i.e. the regulation of vascular relaxation and/or cell redox balance, NO production and vascular smooth muscle dilation. ED comprehends the alteration of fundamental aspects in cell and tissue metabolism, in regulating their homeostasis and development, and in strengthening their ability to react to stress situations. The complexity of this phenomenon involves a large number of actors and effectors and among these, H_2_S emerged as one of the main characters in the regulation of endothelial functionality. Indeed, H_2_S controls the homeostasis of endothelial function and an impairment of its endogenous production is related to the pathogenesis of ED. For this reason, strategies employing compounds able to donate H_2_S could provide in the near future innovative drug candidates for the prevention and treatment of ED in different pathological conditions, such as hypertension, diabetes and atherosclerosis.

Thanks to the intense efforts that have recently been implemented by biomedical research in this field, we presently know that many compounds are actually druggable H_2_S-donors and the searching for molecules that may exhibit a satisfactory and physiological-like H_2_S generation represents a challenging and timely issue. It is noteworthy that several drugs are currently under evaluation in promising clinical studies. Among the several molecules, a very significant part is represented by sulfur compounds of natural origin which could represent a useful pharmacological tool to be used in therapy but could also provide a precious template for the design of new H_2_S-donor molecules with improved pharmacodynamic and/or pharmacokinetic characteristics.

Although the data reported in the literature demonstrate that molecules able to donate H_2_S may represent a useful tool in the protection of ECs in different pathological conditions, further clinical studies are needed. Indeed, there are no specific clinical trials involving receiving H_2_S-donors patients affected by those pathologies that leads to ED. These studies would be very useful, to investigate the reduction, or the slowing down of ED related to specific pathological conditions (diabetes, HHcy, atherosclerosis or hypertension).

## Compliance with Ethics Requirements

This article does not contain any studies with human or animal subjects.

## Funding

This study was supported by the 10.13039/501100003407Italian Ministry of University and Research (MIUR) PRIN 2017XP72RF - Hydrogen Sulfide in the Vascular inflamm-Aging: role, therapeutic Opportunities and development of novel pharmacological tools for age-related cardiovascular diseases (SVAgO).

## Declaration of Competing Interest

The authors declare that they have no known competing financial interests or personal relationships that could have appeared to influence the work reported in this paper.
